# A 6-month sustained-release formulation of triptorelin for locally advanced or metastatic prostate cancer: a real-world experience in Asia

**DOI:** 10.1186/s12894-025-01717-7

**Published:** 2025-02-25

**Authors:** Chi-Hang Yee, Yuen-Hei Chung, Ivan Ching-Ho Ko, Chris Ho-Ming Wong, Alex Mok, Jeremy Yuen-Chun Teoh, Peter Ka-Fung Chiu, Chi-Fai Ng

**Affiliations:** https://ror.org/00t33hh48 grid.10784.3a 0000 0004 1937 0482 S.H. Ho Urology Centre, Department of Surgery, The Chinese University of Hong Kong, Hong Kong, Hong Kong

**Keywords:** Prostate cancer, Hormonal therapy, Androgen deprivation therapy

## Abstract

**Objective:**

Long-acting triptorelin (LAT) (22.5 mg) is a gonadotropin-releasing hormone (GnRH) agonist used in men with prostate cancer. This study investigated the prescription pattern of LAT in a real-world setting and its efficacy.

**Patients & methods:**

This was a retrospective review of patients in a tertiary center who were prescribed LAT for prostate cancer from January 2018 to March 2023 after the introduction of LAT in the territory. Demographic data were collected, and LAT prescription patterns were reviewed. These patterns included the indication and duration of prescription, testosterone suppression and characteristics of the primary prostate cancer.

**Results:**

A total of 237 prostate cancer patients were prescribed LAT in the study period. The indications for LAT included metastatic prostate cancer (50.6%), neoadjuvant/adjuvant therapy for radiotherapy (28.7%) and neoadjuvant therapy for radical prostatectomy (5.1%). Among the cohort, 41.4% of the patients were receiving short-acting triptorelin (11.25 mg) before LAT initiation, 15.2% were receiving other GnRH agonists, and 15.6% were receiving GnRH antagonists. The median age at the first dose of LAT and the median treatment duration were 72 (53–94) years and 30 (6–72) months, respectively. During the study period, 92.0% of the patients did not receive another form of hormonal treatment other than LAT. A total of 121 (51.1%) patients had their testosterone level checked after LAT initiation. The median time interval of testosterone measurement after LAT initiation was 8 (1–47) months, with 98.3% of the patients having a testosterone level < 1.7 nmol/L and 92.6% having a level < 0.7 nmol/L. Among the cohort, 1 patient stopped LAT due to hot flashes and muscle weakness.

**Conclusion:**

The LAT adherence rate was high in the setting of hormonal treatment for prostate cancer. Testosterone suppression was satisfactory after the initiation of LAT and was generally well tolerated.

## Introduction

Prostate cancer is the second most prevalent cancer in men worldwide [1], and its incidence has been increasing in Asia [2]. In recent years, the landscape of advanced prostate cancer management has rapidly changed. Novel options have been shown to improve overall survival in both patients with metastatic hormonal-sensitive prostate cancer (mHSPC) [3–5] and patients with metastatic castration-resistant prostate cancer (mCRPC) [6, 7]. However, androgen deprivation therapy (ADT) with gonadotropin-releasing hormone (GnRH) agonists or antagonists remains the cornerstone of hormonal treatment [8].

ADT aims to reduce testosterone levels to the levels achieved with surgical castration. ADT is recommended for patients with metastatic prostate cancer as well as for those with locally advanced prostate cancer who are unwilling or unable to receive any form of local treatment [9]. Furthermore, ADT has been used in treatment intensification for radiotherapy [10]. Neoadjuvant therapy with ADT before radical prostatectomy tends to lead to a reduction in positive surgical margins, node-positive disease and pathological downstaging [11]. The most widely used ADT in clinical practice is GnRH agonist therapy, and triptorelin is a frequently prescribed agent. Triptorelin potently inhibits gonadotropin secretion when it is administered continuously at therapeutic doses [12]. Triptorelin initially stimulates the anterior pituitary gland, leading to a transient increase in the levels of luteinizing hormone (LH) and follicle stimulating hormone (FSH), resulting in a testosterone surge. However, continuous triptorelin administration leads to receptor desensitization and downregulation, eventually inhibiting gonadotropin secretion. Consequently, serum testosterone levels decrease to the range usually observed following bilateral orchiectomy [12].

Triptorelin is administered to patients in the form of acetate or pamoate (also known as embonate) salts. ADT regimens involving triptorelin can be used in sustained-release formulations for 1 month (3 or 3.75 mg), 3 months (11.25 mg), and 6 months (22.5 mg) [13]. The availability of long-acting triptorelin (LAT) (22.5 mg) with a 6-month injection interval increases convenience and flexibility for patients in need of ADT. This formulation was introduced to our locality in 2018 for patients with prostate cancer. In the present study, we reviewed the characteristics of patients receiving LAT, as well as the usage pattern of LAT in a real-world setting. Furthermore, the efficacy and tolerance of LAT as an ADT option were investigated.

## Methods

This was a retrospective review of consecutive patients who were prescribed LAT (Diphereline® PR 22.5 mg) for prostate cancer from January 2018 to March 2023 in a tertiary referral center. In the setting of our health system, all GnRH agonists and antagonists are covered by government funding, with no costs for patients. The primary objective of the analysis was to explore the usage pattern of LAT in real-world clinical practice. The secondary objectives included determining the proportion of time that testosterone was suppressed below the target level and the prostate‐specific antigen (PSA) kinetics of the study population.

The medical records of patients of Chinese heritage with any of the following prostate cancer statuses were reviewed: (1) high-risk prostate cancer treated with hormonal therapy as neoadjuvant/adjuvant treatment for prostatectomy or radiotherapy; (2) advanced/metastatic prostate cancer treated with long-term hormonal therapy; (3) prostate cancer treated with primary therapy (i.e., surgery or radiotherapy) with biochemical recurrence; and (4) castration-resistant prostate cancer. Furthermore, only patients who received a minimum of one injection of LAT during the subject identification period and had a minimum of 6 months of follow-up data recorded in their medical files were included. Patient electronic records, which included outpatient consultation records, inpatient admission records, drug administration records and laboratory results, were reviewed. The data collected included demographic data, details on prostate cancer, the pattern of hormone use and the testosterone response. Testosterone data were collected at baseline before the initiation of LAT and during the treatment period according to the attending physician’s discretion. The suppression targets for the serum testosterone concentration were < 0.7 nmol/L and < 1.7 nmol/L. PSA data were collected before LAT initiation and toward the end of the study period. mCRPC was defined as prostate cancer that progressed clinically, radiographically, or biochemically despite serum testosterone levels observed after castration. Specifically, CRPC was defined as a documented increase in the PSA level ≥ 2 ng/mL, a PSA level > 25% above nadir, an increase in PSA levels in three consecutive measurements taken at least one week apart, and/or radiological progression in patients who underwent castration and had serum testosterone levels < 1.7 nmol/L (< 50 ng/dL) [14, 15].

We used IBM SPSS Statistics for Windows, version 26.0 (released 2019; IBM Corp., Armonk, New York, United States) for statistical analysis. Continuous data are reported as the means ± standard deviations (SDs) or medians and ranges. A paired t test was used to compare the mean PSA levels before and after LAT administration, and ANOVA was used to analyze the correlation among the duration of LAT treatment, the testosterone level and the eventual change in the PSA level. *p* < 0.05 was considered to indicate statistical significance.

## Results

During the review period, a total of 237 patients with prostate cancer were prescribed LAT. Among these patients, 119 (50.2%) presented with de novo mHSPC, and 160 (67.6%) had an adenocarcinoma of the prostate with a histology of an International Society of Urological Pathology grade group (ISUP GG) of 3 or above (Table 1). Among the patients with metastatic disease, 82 (68.9%) had at least one follow-up radiological assessment. Among these patients, 101 (47.9%) underwent bone scans, 90 (42.7%) underwent a prostate-specific membrane antigen (PSMA) positron emission tomography–computed tomography (PET/CT) and 20 (9.5%) underwent contrast CT of the abdomen and pelvis. The mean interval of restaging radiological assessment was 17.5 ± 13.3 months. During the study period, the all-cause mortality rate was 27.0%. In terms of mortality, disease progression accounted for 43.8% of the cases, and respiratory causes accounted for 28.1%. None of the mortality cases were related to LAT. Among all the patients who died due to disease progression, 75% (7/28) underwent 2 or more radiological assessments after LAT, and 14.3% (4/28) had a PSA level < 50 ng/mL.

**Table 1:** Profile of patients on long-acting triptorelin 22.5mg

Number of patients (n)	237
Prostate cancer metastatic status at diagnosis (n)	
M0	118 (49.8%)
M1a	17 (7.2%)
M1b	84 (35.4%)
M1c	18 (7.6%)
Prostate cancer ISUP GG (n)	
GG 1	28 (11.8%)
GG 2	28 (11.8%)
GG 3	24 (10.1%)
GG 4	44 (18.6%)
GG 5	92 (38.9%)
Local treatment received for prostate cancer (n)	
Nil	88 (37.1%)
Radical prostatectomy	28 (11.8%)
Radiotherapy	106 (44.7%)
TURP	10 (4.2%)
Focal therapy	1 (0.4%)
Others	4 (1.7%)
Median PSA before LAT treatment (ng/mL)	14.3 (0.03 – 12,689)
All-cause mortality during study period (n)	64 (27.0%)

*ISUP GG* International Society of Urological Pathology grade group, *LAT* Long-acting triptorelin, *TURP* Transurethral resection of prostate

The usage patterns of LAT are summarized in Table 2. The study population was a heterogeneous group of patients for whom LAT was used mostly in a metastatic prostate cancer setting (50.6%), together with other indications, including the neoadjuvant/adjuvant setting and locally advanced palliative setting. In addition, 72.2% of the patients had prior exposure to other forms of ADT. As a result, the mean time interval between prostate cancer diagnosis and the first dose of LAT was 26.6 months. Most of the patients received a shorter-acting 3-month triptorelin formulation (11.25 mg) before their treatment was switched to LAT (41.4%), and most of them were in a neoadjuvant/adjuvant setting for radiotherapy (42.9%) or metastatic prostate cancer setting (35.7%) (Table 3). Other forms of GnRH agonists used prior to LAT in this cohort included leuprorelin (Enantone® 11.25 mg, Enantone® 30 mg, Eligard® 22.5 mg, Eligard® 45 mg) and goserelin (Zoladex® 10.8 mg). The switch from other hormonal treatments to LAT was mostly due to a less frequent injection schedule (85.2%), and 92.0% of the patients adhered to the use of LAT as their form of ADT during the review period (Table 2). Except for 1 patient who experienced muscle weakness and hot flashes after LAT use and later stopped ADT and opted for an intermittent ADT regimen, no other documented intolerance to LAT leading to a change in the ADT regimen occurred according to the electronic medical records of all patients. With respect to other side effects, 10 patients (4.2%) experienced fatigue, 8 patients (3.4%) experienced hot flashes, 5 patients (2.1%) experienced night sweats, 4 patients (1.7%) experienced insomnia, and 2 patients (0.8%) experienced cognitive disability. The percentages of patients who started diabetic medication, hyperlipidemic medication and antihypertensive medication during LAT administration were 15.3%, 30.8% and 15.3%, respectively. Among the patients who received 2 or fewer LAT injections before they stopped ADT or changed to other forms of ADT, the most common reasons were death during the review period (33.3%) or the use of LAT as a neoadjuvant treatment before radiotherapy or radical prostatectomy (20.4%) (Table 2).

**Table 2:** Usage pattern of long-acting triptorelin 22.5mg

Indication of LAT (n)			
Metastatic prostate cancer	120 (50.6%)		
Neoadjuvant therapy for radical prostatectomy	12 (5.1%)		
Neoadjuvant / adjuvant therapy for radiotherapy	68 (28.7%)		
Palliative treatment for localized prostate cancer	30 (12.6%)		
Recurrence after initial radical treatment	7 (3.0%)		
	**Metastatic CaP**	**Localized CaP**	**Overall**
Median age of 1st dose of LAT (year)	70 (53 – 94)	72 (53 – 94)	72 (53 – 94)
Mean time interval between diagnosis and 1st dose LAT ± SD (m)	21.4 ± 36.7	19.2 ± 29.7	26.6 ± 99.5
Median duration of LAT treatment (m)	32 (6 – 74)	27 (6 – 74)	30 (6 – 72)
Hormonal treatment before commencement of LAT (n)			
Nil	36 (25.0%)	30 (25.6%)	66 (27.8%)
3-monthly formulation shorter-acting triptorelin 11.25mg	32 (26.7%)	66 (56.4%)	98 (41.4%)
Other forms of GnRH agonist	22 (18.3%)	14 (12.0%)	36 (15.2%)
GnRH antagonist	30 (25.0%)	7 (6.0%)	37 (15.6%)
Median duration of ADT before commencement of LAT (m)			
3-monthly formulation shorter-acting triptorelin. 11.25mg	3 (3—33)	6 (3—58)	3 (3—58)
Other forms of GnRH agonist	12 (6—78)	6 (6—56)	12 (6—78)
GnRH antagonist	5 (1—58)	2 (1—43)	3 (1—58)
Reason for changing from other ADT to LAT (n)			
Less frequent injection schedule	100 (83.3%)	102 (85.0%)	202 (85.2%)
Physician’s preference	20 (16.7%)	15 (15.0%)	35 (14.8%)
Change of hormonal treatment during study period (n)			
Nil	108 (90.0%)	110 (94.0%)	218 (92.0%)
3-monthly formulation shorter-acting triptorelin 11.25mg	2 (1.7%)	1 (0.9%)	3 (1.2%)
Other forms of GnRH agonist	7 (5.8%)	2 (1.7%)	9 (3.8%)
GnRH antagonist	3 (2.5%)	4 (3.4%)	7 (3.0%)
Reason for changing from LAT to other hormonal treatment (n)			
Cardiovascular risk (to GnRH antagonist)	2 (16.7%)	2 (28.6%)	4 (21.1%)
Physician’s preference	4 (33.3%)	4 (57.1%)	8 (42.1%)
Patient’s preference	2 (16.7%)	1 (14.3%)	3 (15.8%)
Others	4 (33.3%)	0 (0.0%)	4 (21.1%)
Patients on LAT but for < / = 2 doses (n)	19 (8.0%)	35 (14.8%)	54 (22.8%)
Reason for not continuing LAT after 1 or 2 doses			
Cardiovascular risk (to GnRH antagonist)	2 (10.5%)	2 (5.7%)	4 (7.4%)
Patient passed away	11 (57.9%)	7 (2.0%)	18 (33.3%)
As neoadjuvant treatment	0 (0.0%)	11 (31.4%)	11 (20.4%)
Physician’s preference	2 (10.5%)	3 (8.6%)	5 (9.3%)
Patient’s preference	1 (5.3%)	0 (0.0%)	1 (1.9%)
Patient defaulted follow-up	2 (10.5%)	6 (17.1%)	8 (14.8%)
Side-effects	0 (1.9%)	1 (2.9%)	1 (1.9%)
Others	1 (5.3%)	5 (14.3%)	6 (11.1%)

*ADT* Androgen deprivation therapy, *CaP* Prostate cancer, *GnRH* Gonadotrophin-releasing hormone, *LAT* Long-acting triptorelin

**Table 3:** Disease stage of patients when 3-monthly triptorelin 11.25mg was changed to long-acting triptorelin 22.5mg

Number of patients (n)	98
Metastatic prostate cancer (mHSPC) (n)	35 (35.7%)
Neoadjuvant therapy for radical prostatectomy (localized prostate cancer) (n)	8 (8.2%)
Neoadjuvant / adjuvant therapy for radiotherapy (localized prostate cancer) (n)	42 (42.9%)
Palliative treatment for localized prostate cancer (n)	8 (8.2%)
Recurrence after initial radical treatment (localized prostate cancer) (n)	5 (5.1%)

*mHSPC* Metastatic hormonal sensitive prostate cancer

In the series, 63 (26.6%) patients developed mCRPC; among these patients, 19 (30.2%) received docetaxel, 27 received androgen receptor-targeted agents (ARTAs), 6 (9.5%) received Lutetium 177, and 2 (3.2%) received Radium 223. Among the patients who did not develop mCRPC, 6 (3.5%) received docetaxel, and 21 (12.2%) received ARTAs. In the whole cohort, 51.1% of the patients had their testosterone measured during LAT use. The median time between the initiation of ADT and the first testosterone measurement after LAT initiation was 17 (1–54) months, and the median time between the initiation of LAT and the first testosterone measurement after LAT initiation was 8 (1–47) months (Table 4). Among the patients with testosterone data, 79 (65.3%) had metastatic prostate cancer. Overall, 98.3% of the patients achieved a testosterone level < 1.7 nmol/L, and 92.6% of the patients achieved a testosterone level < 0.7 nmol/L. Compared with that of the patients receiving ADT during the same period, the proportion of patients receiving LAT who achieved testosterone levels < 0.7 nmol/L did not significantly differ (Table 4). A statistically significant difference in PSA levels was detected before the initiation of LAT and at the end of the review period (286.4 ± 1638.0 ng/mL to 57.8 ± 213.8 ng/mL, *p* < 0.01). Multivariate analysis did not reveal a correlation among the duration of LAT use, testosterone level and eventual change in the PSA level (F(1,70) = 0.24, *p* = 0.63).

**Table 4:** Testosterone level, PSA level and castration-refractory prostate cancer status during study period

Patients with testosterone level checked after LAT (n)	121 (51.1%)	
Median time interval of testosterone checked after LAT (month)	8 (1 – 47)	
Patients with testosterone reaching castration level after LAT (n)		
Testosterone < 1.7 nmol/L	119 (98.3%)	
Testosterone < 0.7 nmol/L	112 (92.6%)	
Comparison of LAT with other ADT in the same period		
Leuprolide with testosterone < 0.7 nmol/L	124/141 (87.9%)	*p* = 0.213
LHRH antagonist with testosterone < 0.7 nmol/L	90/101 (89.1%)	*p* = 0.371
Mean PSA before LAT treatment (ng/mL)	286.4 ± 1638.0	
Mean PSA in the end of study period with LAT (ng/mL)	57.9 ± 213.8	*p* < 0.01
mCRPC status (n)		
In mCRPC status before starting LAT	2 (0.8%)	
Developed mCRPC status during LAT	63 (26.6%)	
Not in mCRPC status during study period	172 (72.6%)	
Mean time to develop CRPC status (months)		
From the start of ADT	42.5 (5.0 – 86.0)	
From the start of LAT	17.5 (3.0 – 74.0)	

*ADT *Androgen deprivation therapy, *mCRPC *Metastatic castration-refractory prostate cancer, *LAT *Long-acting triptorelin, *LHRH *Luteinizing hormone-releasing hormone, *PSA *Prostate specific antigen

## Discussion

While ADT is the backbone of hormonal therapy for metastatic prostate cancer, our study revealed that a significant number of patients received ADT in a nonmetastatic disease setting. Almost 50% of the patients receiving LAT in our review did not have metastatic prostate cancer. This finding illustrates that the impact of ADT extends beyond the metastatic population. Furthermore, even though neoadjuvant ADT is not currently recommended routinely before radical prostatectomy, 5.1% of the patients receiving LAT were prescribed it for this reason. In other national surveys, we reported that the incidence rate of neoadjuvant ADT use before radical prostatectomy ranged from 2.1% to 15.4% [16, 17]. This highlights the role of real-world data in providing another perspective for evaluating the usage of ADT.

The biochemical effectiveness of ADT is measured by determining whether testosterone levels are reduced by treatment to levels observed after castration. The historical testosterone cutoff value after castration is < 1.7 nmol/L (< 50 ng/dL), which is based on early tests that can detect testosterone only above this level. However, with the improved sensitivity of modern assays, the mean testosterone level following bilateral orchiectomy is 0.5 nmol/L (15 ng/dL) [18]. A lower testosterone cutoff of < 0.7 nmol/L (< 20 ng/dL) resulted in improvements in survival-related outcomes [19]. In an open-label equivalence study, 96% and 98% of the patients receiving the 1- and 3-month formulations of triptorelin achieved a plasma testosterone level < 1.7 nmol/L [20]. For the 6-month formulation of LAT, 97.5% of patients achieved a serum testosterone level < 1.7 nmol/L by day 29 after castration in a phase III trial of patients with advanced prostate cancer [21]. With respect to the more stringent target of a testosterone level < 0.7 nmol/L after castration, a pooled analysis revealed that > 90% of patients receiving 1-, 3- or 6-month formulations of triptorelin achieved serum testosterone levels < 20 ng/dL at months 3, 6, 9 and 12 [22]. Furthermore, when comparing goserelin, triptorelin and leuprolide, the testosterone level was found to be maintained below the castration level over a longer duration for > 90% of the patients receiving LAT (testosterone level < 0.7 nmol/L for 48 weeks). In the present review, the testosterone level was below < 0.7 nmol/L in 92.6% of the patients at a median time interval of 8 months after LAT initiation and a median time interval of 17 months after ADT initiation. The ability of testosterone suppression is similar for LAT in the real-world setting of an Asian population.

Our review revealed that while there is a wide range of dosing frequencies for ADT injections, less frequent injections are an incentive for patients and physicians to switch to a new formulation. In the present cohort, 85.2% of the patients were switched to LAT from another form of ADT for this reason. Indeed, it has been reported that patients with prostate cancer prefer 3- or 6-month dosing regimens rather than 1-month regimens, which aligns with the usual monitoring frequency in different institutes. Furthermore, these formulations have been shown to result in reduced annual costs compared with 1-month formulations [23]. In a study conducted in France, it was found that a switch from a 3-month to a 6-month formulation of ADT occurred more often than a switch from a 6-month to a 3-month formulation, with various reasons stated, including therapy simplification and avoidance of unnecessary visits to the doctor [24]. In our institution, the follow-up interval for patients with prostate cancer is usually 6 months. This leads to an additional advantage of 6-month formulations, which allow for a synchronized injection and follow-up schedule. As a result, convenience for patients and physicians is one of the critical factors to consider choosing from a group of comparable hormonal therapy options.

In real-world settings, the cost of medication and stock availability are other factors that play a role in treatment decisions. In our institute, the cost of ADT injections was covered only by the government and was thus free to patients after 2019. Previously, different forms of ADT had some variation in their price for patients. As a result, patients may make decisions on the basis of financial concerns. Furthermore, physicians’ own habits and preferences play an important role in their prescription practices. In our review, many physicians preferred starting the 3-month triptorelin formulation before continuing with the 6-month formulation LAT later. This practice may facilitate an earlier review of the patient after starting ADT and the detection of any side effects from treatment, if present. These considerations explain why some of the patients switched from one form of ADT to another during our review period.

In general, LAT has been reported to be well tolerated in trials including patients with locally advanced or metastatic prostate cancer [12]. The most common adverse events were hot flashes and decreased libido, which are common among different GnRH agonists. Only 1 patient in our cohort stopped LAT due to side effects. Using the criterion of 2 or fewer injections of LAT as a surrogate marker to identify potential patients with intolerable side effects from LAT, we did not identify additional patients who stopped LAT due to side effects. We may conclude that LAT was generally well tolerated in our Asian cohort and that the need to stop the medication due to side effects was rare. Indeed, a recent study of Asian patients with prostate cancer also revealed that the Asian population was quite tolerant to ADT in general, with no change in the EuroQoL visual analog scale (EQ-VAS) score from baseline to 12 months [25]. During the review period, some patients were switched from LAT to a GnRH antagonist because of their cardiovascular risk. A systematic review by Nelson et al. comparing the cardiovascular effects of GnRH antagonists and agonists suggested that the use of GnRH antagonists may be associated with fewer cardiovascular events [26]. Crawford et al. recently reviewed the data in the Clarivate Real World Evidence repository, analyzing data from 45,050 men [27]. The authors reported that the adjusted incidence of major adverse cardiovascular events was greater for men treated with GnRH antagonists than for those treated with GnRH agonists. Further investigations are needed to address this issue, as calculating the cardiovascular risk of patients receiving ADT is complex and depends on the baseline cardiovascular profile, concomitant metabolic factors, lifestyle habits, treatment outcomes and sequence [28].

The present review is limited by its retrospective nature. As a result, standardized radiological or histological reassessment for evaluating treatment response and disease activity is lacking. Furthermore, the duration of exposure to LAT differed between the subjects, which could confound the assessment of LAT efficacy and adverse effects. Moreover, standardized assessments of side effects of LAT by using validated questionnaires as well as a comprehensive testosterone profile of the cohort were missing. However, adherence to treatment and testosterone levels in the mCRPC setting could be used as surrogate markers to reflect the effectiveness and tolerance of LAT. Furthermore, our study was limited by the heterogeneous ADT profiles of the patients, with most patients having prior exposure to other forms of GnRH agonists or antagonists. This would impair the assessment of testosterone and PSA responses to LAT. However, our review may illustrate the possible interplay between different ADT regimens in the real-world clinical setting.

## Conclusion

The use of ADT extends from localized prostate cancer to metastatic disease. In real-world clinical settings, 6-month LAT has shown clinical efficacy because of its testosterone suppression, tolerability because of its favorable side-effect profile, and potential convenience because of its relatively long injection interval. Further investigations are needed to delineate the role of different options in the ADT spectrum.

## Data Availability

The datasets generated and/or analysed during the current study are not publicly available due to confidentiality concern but are available from the corresponding author on reasonable request.
